# Intraguild Predation Responses in Two Aphidophagous Coccinellids Identify Differences among Juvenile Stages and Aphid Densities

**DOI:** 10.3390/insects5040974

**Published:** 2014-12-08

**Authors:** Gabriele Rondoni, Fulvio Ielo, Carlo Ricci, Eric Conti

**Affiliations:** Department of Agricultural, Food and Environmental Sciences, University of Perugia, Borgo XX Giugno 74, Perugia 06121, Italy; E-Mails: fulvio.ielo@gmail.com (F.I.); carlo.ricci@unipg.it (C.R.); eric.conti@unipg.it (E.C.)

**Keywords:** *Aphis gossypii*, biological control, *Coccinella septempunctata*, competitive interactions, *Hippodamia variegata*

## Abstract

(1) Intraguild predation (IGP) can occur among aphidophagous predators thus reducing their effectiveness in controlling crop pests. Among ladybirds, *Coccinella septempunctata* L. and *Hippodamia variegata* Goeze are the most effective predators upon *Aphis gossypii* Glov., which is an economically important pest of melon. Understanding their likelihood to engage in reciprocal predation is a key point for conservation of biological control. Here, we aim to investigate, under laboratory conditions, the level of IGP between the two above mentioned aphidophagous species. (2) Fourth-instars of the two species were isolated in petri dishes with combinations of different stages of the heterospecific ladybird and different densities of *A. gossypii*. The occurrence of IGP events was recorded after six hours. (3)* C. septempunctata* predated *H. variegata* at a higher rate than *vice versa* (70% *vs.* 43% overall). Higher density of the aphid or older juvenile stage of the IG-prey (22% of fourth instars *vs.* 74% of eggs and second instars) reduces the likelihood of predation. (4) To our knowledge, IGP between *C. septempunctata* and *H. variegata* was investigated for the first time. Results represent a baseline, necessary to predict the likelihood of IGP occurrence in the field.

## 1. Introduction

Generalist predators, including coccinellids, are very effective in pest suppression [1]. In fact, they colonize agricultural fields early in the season by feeding on alternative prey [2] and thus increase their populations prior to the arrival of the pest [3]. However, coccinellids can, under certain conditions, engage in competitive interactions between members of the same trophic level [4]. Intraguild predation (IGP) occurs where a predator (IG-predator) consumes another species (IG-prey) that shares a common prey, and has been commonly reported among coccinellids [5,6,7,8,9,10,11,12]. Predation on guild members by a top predator has been implicated as the primary cause for displacement of some selected species [13] and, in some cases, to disrupt biological control [14].

*Aphis gossypii* Glov. has a worldwide distribution and is a major economically important pest of Cucurbitaceae, including melon [15,16]. As a consequence of its feeding activity, the aphid causes leaves to curl up, damaging seriously the vegetation and fruits. In addition, the aphid is a vector of plant pathogen viruses, such as the Cucumber Mosaic Virus [17]. Control measures include chemical treatments and the use of resistant varieties, but due to the ability of *A. gossypii* to develop resistance to insecticides [18,19] and to overcome plant genetic defenses, these two methods do not guarantee efficient control [20]. Among cultural methods, UV-absorbing films used in tunnels or mulching has been shown to partially protect the crop from *A. gossypii* infestations [21]. Previous studies [22,23,24,25] and our surveys conducted during 2013 in melon crop planted in Central Italy suggest that coccinellids, mostly *Coccinella septempunctata* L. and *Hippodamia variegata* (Goeze), may provide an efficient control over the population of this pest. However, before considering these natural enemies for conservation biological control purposes, it is necessary to determine their likelihood to establish intraguild interactions, with unpredictable consequences for disruption of aphid biological control (reviewed by [7,26]). Therefore, the objective of this study was to determine, under laboratory conditions, the likelihood of intraguild predation between different stages of the two ladybirds at different densities of their essential prey, *A. gossypii*. Coccinellids are vulnerable to IGP during their entire life cycle, but the risk of being IG-prey is stage-dependent and seems to be higher with low density of aphids (reviewed by [27]). In particular, we test the hypothesis that ladybird beetle eggs and early juvenile stages are more susceptible to IGP than older stages, and that the IGP levels are lower at higher aphid densities.

## 2. Experimental Section

### 2.1. Insect Rearing and Experimental Set-Up

Coccinellid cultures were established from *C. septempunctata* and *H. variegata* adults collected from melon crop field in Central Italy and reared on an *ad libitum* diet of *Aphis fabae* Scopoli. *Aphis gossypii* winged females were collected from the field and populations were established in the laboratory on *Hibiscus syriacus* L. plants.

Fourth-instars of either *C. septempunctata* or *H. variegata* (within 8 h after ecdysis) were isolated in 15 mL glass test tubes (1.5 cm diameter), containing a strip of filter paper, closed with cotton wool and arranged horizontally. Larvae to be screened as IG-predator were starved for 12 h (water provided on cotton wool) to induce a constant level of hunger [28]. Subsequently, each coccinellid larva was randomly transferred to a clean petri arena (9 cm diameter), containing a moistened filter paper on the bottom, and allowed to feed on a combination of (i) one egg or (ii) a second-instar or (iii) a fourth-instar of a heterospecific coccinellid (*i.e*., *H. variegata* if the starved larva was *C. septempunctata* and *vice versa*) with, respectively, 0, 5, 10, 25, 50, 100 or 200 *A. gossypii* individuals. Filter papers were moistened in order to provide a good environment for larvae and to avoid insects from hiding below the paper. Whether or not each starved larva completely ate the IG-prey item was recorded after six hours. The larvae were further allowed to feed for additional 18 h to ensure they were able to entirely consume during this interval the amount of aphids provided. The experiment was replicated seven times for each combination of IG-predator, IG-prey and aphid density, with a total of 294 observations. Insect rearing and experiments were performed in a controlled environmental chamber at 25 ± 1 °C, 70% ± 5% RH, and 14 h L:10 h D photoperiod.

### 2.2. Data Analysis

Logistic regression was performed to analyze the relationship between IGP frequency and the variables: IG-predator species, IG-prey stage, initial aphid density, and the interactions between them. To account for data separation, logistic regression models were fitted by applying Firth’s correction [29,30,31], which allowed us to obtain finite parameter estimates for the predation response of *C. septempunctata* to eggs at different aphid densities. To identify the minimum adequate model, we used backward elimination, starting with a model that included the IG-predator species, initial aphid density and IG-prey stages and all the interactions between variables. Model selection was performed based on penalized likelihood ratio test [30]. All data analyses were performed under R statistical environment [32], with package “logistf” used to perform Firth’s penalized likelihood logistic regression [33].

## 3. Results and Discussion

The best-fitted model retained the principal effect of the IG-predator species, the IG-prey juvenile stage and the aphid density, without any interactions between them (Table 1).

**Table 1:** Regression coefficients, standard errors and significance for the variables retained in the best fitted models describing the relationship between the frequency of IGP and: (i) the IG-predator species (*Coccinella septempunctata* or *Hippodamia variegata*); (ii) the IG-prey stage (egg, second- or fourth-instar); (iii) the initial density of aphids.

Variable	(Level)	Coeff	SE (Coeff)	*p*-Value
Intercept		4.208	0.530	<0.001
IG-predator species	(*H. variegata*)	−1.864	0.350	<0.001
IG-prey stage	(Second-instar)	−2.119	0.439	<0.001
	(Fourth-instar)	−4.182	0.507	<0.001
Aphid density		−0.012	0.003	<0.001

**Figure 1:**
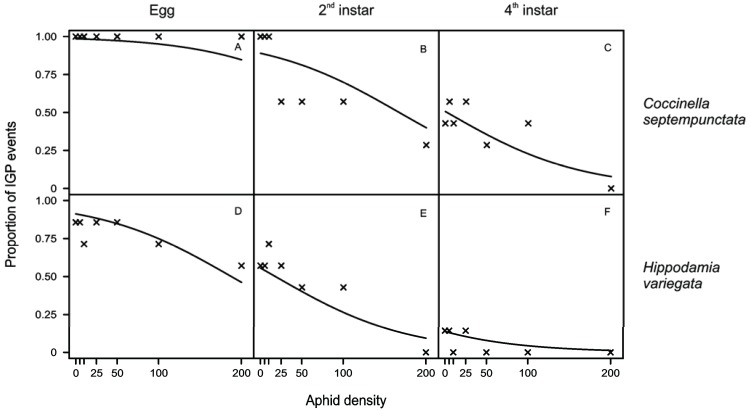
Proportion of IGP events by *Coccinella septempunctata* L. (**A**–**C**) and *Hippodamia variegata* (Goeze) (**D**–**F**) upon egg (**A**,**D**), second-instar (**B**,**E**) and fourth-instar (**C**,**F**) of the IG-prey at different density of *Aphis gossypii* Glov. The symbols represent the proportion of the replicates in which IGP occurred, while the line indicates penalized maximum likelihood estimate of the logistic response function.

IGP probability was significantly higher with *C. septempunctata* (Figure 1A–C, overall 70% of replicates with IGP events), rather than with *H. variegata* (Figure 1D–F, IGP in 43% of replicates) and was significantly reduced with the increase of the initial aphid density (overall in more than 52% of replicates with less than 100 aphids provided). Eggs (Figure 1A,D) were more susceptible to IGP than larvae (overall 89% of predated eggs *vs.* 41% of larvae), with second-instars (Figure 1B,E) more likely to be predated upon than fourth-instars (Figure 1C,F). All individuals consumed the whole amount of aphids provided within 24 h. 

## 4. Discussion

Our results revealed that *C. septempunctata* may act as a strong predator of *H. variegata*, in particular upon early juvenile stages, and this corroborates previous findings on other IG coccinellid preys (reviewed by [27]). *Coccinella septempunctata* larvae demonstrated asymmetric predation upon *Adalia bipunctata* (L.), both in petri arena [10] and in semi-field experiments [34]. Snyder, *et al.* [35], under laboratory conditions, reported that both *Coccinella transversoguttata* Brown and *Hippodamia convergens* Guérin-Méneville, dominant native species in agricultural fields in Eastern Washington and Northern Idaho, were often the prey of *C. septempunctata*.

The vulnerability of a species to IGP is dependent on size, with the largest species more likely to act as a IG-predator [27]. In our case the asymmetric predation of *C. septempunctata* upon *H. variegata* larvae could be explained by the higher dimension of larval instars of the former species, although other behavioral aspects should be studied (*i.e*., reciprocal attack and escape rates, defensive behavior [28,36]). Asymmetric predation upon eggs instead, could be explained by the different voracity of the two species [37] or by the different effectiveness of the defensive alkaloids they possess. Coccinellid eggs present chemical compounds, which might protect them from IGP [38,39]. In this respect, the ability of an IG-predator to detoxify heterospecific chemical compounds after the ingestion might, in part, explain its aggressive behavior [40] and further studies are needed to verify the different tolerance of *C. septempunctata* and *H. variegata* to heterospecific eggs’ consumption. Our results does not necessarily mean that *H. variegata* is more at risk in agricultural habitats as a result of the interactions with *C. septempunctata*, and our IGP experiments should be carefully interpreted before determining characteristics of predation in open field systems. In fact, in field conditions, behavioral mechanisms, such as escaping behavior or prey switching, may occur to prevent IGP [41]. Moreover in interpreting our results it is important to consider the different biology and ecology of the two coccinellid species. *Coccinella septempunctata* generally completes one or two generations with females that start to oviposit early in the season [42,43] by laying a huge amount of eggs on herbaceous crops. *Hippodamia variegata*, instead, is a multivoltine species, which completes two to three generations in Europe, thus its reproductive period exceed that of *C. septempunctata* and its population density increases over the season [44,45]. For that reason *H. variegata* seems more abundant in those crops that are harvested late, preventing IGP interactions to some extent. As an example, in Greece, Kavallieratos, *et al.* [46] revealed that *C. septempunctata* was the dominant species on durum wheat (during April–May), while *H. variegata* was predominant on cotton (in June–July). In addition to voltinism, the abundance of the two species could be explained for example by their fecundity (higher in *C. septempunctata* [47]), and by demographic regulation exhibited by natural enemies [48]. When co-occurring on the same plant, the coexistence of the two species could be fostered by a different choice of the oviposition site. Females of both species prefer to oviposit close to aphid’s colonies, however *H. variegata* prefers a more humid microclimate to lay eggs [49]. Moreover, *C. septempunctata* is more likely to oviposit over rough substrates, *i.e*., the lower page of the leaves [50]. Finally, to reduce the likelihood of IGP, ladybird ovipositing females have been shown to be able to detect semiochemicals released from potential competitors and reduce the amounts of eggs laid accordingly [51].

The useful and predictive potential of two-dimensional arena experiments has been demonstrated recently for another species, *Harmonia axyridis* (Pallas). This ladybird acted as asymmetric IG-predator under laboratory conditions [8,34,52,53,54,55,56,57] and demonstrated its aggressiveness also in field systems [58,59,60,61,62,63]. Whether or not the asymmetric predation revealed by *C. septempunctata* over *H. variegata* in small arenas might be predictive of field situations remains to be elucidated. In addition, our results suggest that IGP could be relevant also at higher aphid density. Recently it has been demonstrated that IGP among coccinellids could be common in nature even at high aphid densities [56], and our results on predation of eggs and young larval instars support this. In the context of biological control, considering that *H. variegata* has been proposed as a candidate for augmentative programs against *A. gossypii* [64,65], our results might predict low non-target risks for local *C. septempunctata* populations wherever the two ladybirds co-occur. Conservation approaches might consider the seasonal movement of *C. septempunctata* from arable to vegetable crops [66]. In this respect, the presence of wheat fields close to melon crops could favor the movement of new *C. septempunctata* adults in the vegetable early after the maturation of the cereal in June [67] and this might be useful for an early control upon the aphid colonies.

## 5. Conclusions

In conclusion, our study has value in evaluating the potential of IGP between *C. septempunctata* and *H. variegata* when they share a common prey, the aphid *A. gossypii*. We are presently conducting field investigations on the predator guild that regulates *A. gossypii* populations in melon crop and we are developing molecular gut content analyses on field-collected individuals. In this respect, our results would represent a baseline to predict the likelihood of predation in the field and investigate trophic relationships in coccinellid assemblages and their role in pest suppression.
